# Mirk/dyrk1B Kinase in Ovarian Cancer

**DOI:** 10.3390/ijms14035560

**Published:** 2013-03-08

**Authors:** Eileen Friedman

**Affiliations:** Pathology Department, Upstate Medical University, 750 East Adams Street, Syracuse, NY 13210, USA; E-Mail: friedmae@upstate.edu; Tel.: +1-315-464-7148; Fax: +1-315-464-8419

**Keywords:** Mirk/dyrk1B, quiescence, ROS control

## Abstract

Mirk/dyrk1B kinase is expressed in about 75% of resected human ovarian cancers and in most ovarian cancer cell lines with amplification in the OVCAR3 line. Mirk (minibrain-related kinase) is a member of the Minibrain/dyrk family of related serine/threonine kinases. Mirk maintains cells in a quiescent state by stabilizing the CDK inhibitor p27 and by inducing the breakdown of cyclin D isoforms. Mirk also stabilizes the DREAM complex, which maintains G0 quiescence by sequestering transcription factors needed to enter cycle. By entering a quiescent state, tumor cells can resist the nutrient deficiencies, hypoxic and acidic conditions within the tumor mass. Mirk maintains the viability of quiescent ovarian cancer cells by reducing intracellular levels of reactive oxygen species. CDKN2A-negative ovarian cancer cells treated with a Mirk kinase inhibitor escaped G0/G1 quiescence, entered cycle with high ROS levels and underwent apoptosis. The ROS scavenger N-acetyl cysteine reduced the extent of cancer cell loss. In contrast, the Mirk kinase inhibitor slightly reduced the fraction of G0 quiescent diploid epithelial cells and fibroblasts, and the majority of the cells pushed into cycle accumulated in G2 + M. Apoptotic sub-G0/G1 cells were not detected. Thus, normal cells were spared because of their expression of CDK inhibitors that blocked unregulated cycling and Mirk kinase inhibitor-treated normal diploid cells were about as viable as untreated controls.

## 1. Introduction

A subpopulation of tumor cells, possibly including stem cells, remains in a non-dividing quiescent state that renders them relatively resistant to chemotherapy and radiation therapy that target dividing cells [1]. By entering a quiescent state, tumor cells can resist the nutrient deficiencies, hypoxic and acidic conditions within the tumor mass. Mirk maintains the viability of quiescent ovarian cancer cells by reducing intracellular levels of reactive oxygen species (ROS) [2].

Normal cells in quiescence activate pathways that protect them from metabolic stress, and a subpopulation of tumor cells is likely to utilize similar pathways to survive within the tumor microenvironment. The anti-apoptotic proteins BCL2 and BCL-XL have been shown by others to facilitate G0 quiescence in murine embryonic fibroblasts by decreasing RNA content and cell size, and by upregulating p27 protein through its stabilization following phosphorylation of p27Ser10 by Mirk [3]. Factors that allow the prolonged survival of quiescent tumor cells *in vivo* are of clinical relevance. These include the ras-related tumor suppressor gene *ARH1* which induces autophagy, inhibits the PI3-kinase pathway and regulates dormancy in ovarian cancer cell xenografts [4,5], the stress-activated protein kinase p38 [6], and antioxidant proteins and factors which control their expression [7] such as Mirk, which decreases the level of toxic ROS in tumor cells, increasing their survival [8] and their clonogenic growth [9]. *Mirk* was found to be among the four most promigratory genes in the SKOV3 ovarian cancer cell line [10], suggesting that Mirk might play a role in ovarian cancer spread. Enhanced tumor cell survival during a quiescent period might increase the size of the population of solitary cells capable of spread. The role of Mirk in achieving and maintaining G0 quiescence, as a part of the dormant cell phenotype, was examined in ovarian cancer cells.

## 2. Results and Discussion

### 2.1. *Mirk* Gene Amplification

Amplicons are maintained in cancers when one or more genes within the amplicon provide a selective growth or survival advantage. The 19q13 amplicon was identified in about 30% of ovarian cancers in early studies [11]. Amplifications at 19q13.12 and 19q13.2 were also seen in a recent analysis of 489 high-grade serous ovarian adenocarcinomas [12]. The *Mirk* gene was one of 16 genes comprising the consistently amplified 660 kb subregion of the 19q13 amplicon in pancreatic cancers, while the nearby gene *Akt2* was not [13]. Southern blotting was performed on seven ovarian cancer cell lines that expressed Mirk protein by western analysis. Three of these lines, OVCAR3, SKOV3 and OVCAR8, had a homogenously staining region of amplified genes including *Akt2* at 19q13 [11,14], but only OVCAR3 cells exhibited a 20-fold amplification of the *Mirk* gene [15]. The *p21cip1* gene located on chromosome 6 was used as an internal blotting control as this locus was not amplified or deleted in any ovarian cancer case examined in the NIH database.

### 2.2. Mirk Kinase

Mirk (minibrain-related kinase) is a member of the Mirk/dyrk family of related serine/threonine kinases in eukaryotes and the Minibrain family in fly. The Mirk protein has a conserved kinase domain, unique *N*-terminal and *C*-terminal regions, and a bipartite nuclear localization sequence in its *N*-terminus [16]. Mirk/dyrk1B had endogenous kinase activity when immunoprecipitated from ovarian cancer cell lines [15]. Mirk expression is low in most normal tissues except skeletal muscle, and is elevated in several solid tumor types as well as ovarian cancer.

Mirk/dyrk1B was expressed in 21 of 28 (75%) resected human ovarian cancers, primarily papillary serous cystadenocarcinomas [15]. Analysis of paired samples of cancer and adjacent normal epithelium showed a statistically significant upregulation in 60% of the cancers with *p* = 0.0001 by the student’s paired *t* test [15]. In a similar, but larger, clinical screen of 76 patient samples, including 38 serous adenocarcinomas, 13 mucinous carcinomas, 16 benign cystadenomas and 9 non-neoplastic ovarian cysts, Mirk protein was detected in 75% of the cancers and overexpressed in 41%, with lower incidence in the benign tumors and none in the non-neoplastic ovarian cysts [17]. Mirk/dyrk1B was expressed in each of 7 ovarian cancer cell lines [15] and in 5 of 8 ovarian cancer cell lines [17], again showing frequent expression in this cancer.

### 2.3. Mirk Kinase Function

**Figure 1:**
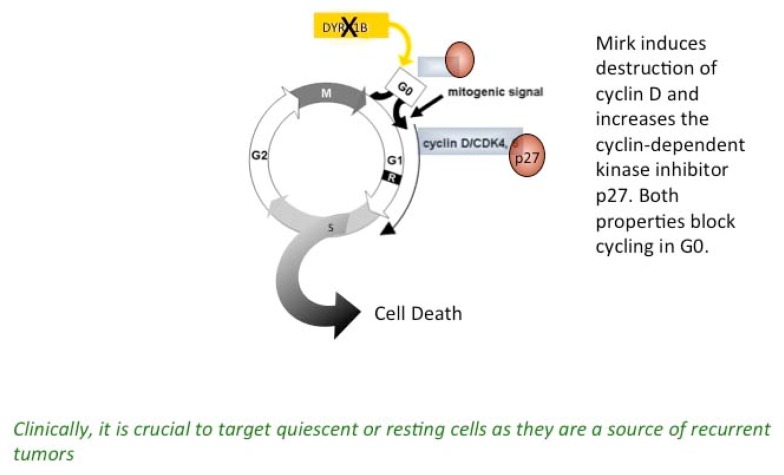
Mirk/dyrk1B regulates G0 quarantine of cancer cells.

**Figure 2:**
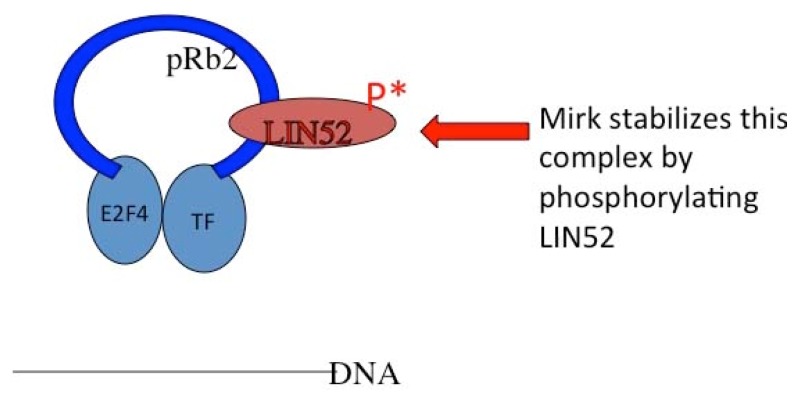
p130/Rb2 holds cells in G0 by binding specific transcription factors.

Mirk functions to maintain normal diploid cells in a quiescent state by stabilizing the CDK inhibitor p27 [18] and by inducing the breakdown of cyclin D isoforms. Mirk binds to GSK3 β, and the complex phosphorylates cyclin D at two adjacent conserved ubiquitination sites, Mirk at T288, and GSK3β at T286 [19]. The entire family of Dyrk family kinases has been implicated in cell cycle regulation, with Dyrk1A and HIPK2 targeting cyclin D1 and p27, respectively [20]. A summary of Mirk kinase function is depicted in the model shown below (Figure 1):

Mirk also phosphorylates LIN52, stabilizing the DREAM complex that maintains G0 quiescence through sequestering transcription factors needed to enter cycle [21], as shown in the model below (Figure 2). Mirk phosphorylates these proteins (LIN52, p27 and cyclin D isoforms) to arrest cycling myoblasts in a G0/G1 state where they can cease cycling and fuse to form multinucleated myotubules and then organize into muscle fibers [22,23]. Mirk depletion prevented this growth arrest and subsequent differentiation.

### 2.4. Mirk Kinase Function

Inactivation of FoxO transcription factors occurs when they are translocated out of the nucleus. Mirk depletion was reported to lead to nuclear translocation of FoxO1 and FoxO3A in ovarian cancer cells, and well as increased apoptosis, suggesting that control of FoxO function through localization is mediated through Mirk kinase, at least in part [17]. It has also been reported that knockdown of Mirk/Dyrk1B by siRNA led to up-regulated activation of the c-Raf-MEK-ERK1/2 pathway and increased culture growth rate, with cells entering from G0/G1 into S phase [24]. This cell cycle movement could be blocked by U0126 in a dose-dependent manner, indicating Mirk/Dyrk1B may sequester the MAPK/ERK pathway, and vice versa. Furthermore, combined Mirk siRNA and U0126 induced cell apoptosis in the human cancer cells. Thus, Mirk/Dyrk1B may also mediate cell cycling and survival via interacting with the MAPK/ERK signaling cascade.

### 2.5. Mirk Kinase Activation

Mirk kinase is not activated by mutation, and no mutations that would alter kinase activity have been found by sequencing solid tumors. Mirk is activated by an oncogenic K-ras or oncogenic H-ras signaling cascade through Rac1 to MKK3 [9]. Mirk is also activated by stress signaling through MKK3, initiated by such chemotherapeutic drugs as 5-fluorouracil and nocodazole. Mirk also competes with the stress kinase p38 for access to their common activating kinase, MKK3.

### 2.6. Mirk Has Two Major Functions *in vivo*, Growth Arrest in a Quiescent G0/G1 State and Maintenance of Viabilit

Mirk depletion also led to a loss of viability in the fused myotubules [23]. Mirk protein is expressed at high levels in normal skeletal muscle and is localized in the fast-twitch fibers that release reactive oxygen species when they contract. Mirk depletion increased ROS levels in each of four ovarian cancer cell lines, suggesting that Mirk expression was upregulated in ovarian cancer cells because it enabled ovarian cancer cells to survive the increase in ROS commonly seen in transformed cells [2].

### 2.7. Both Normal Diploid Cells and Ovarian Cancer Cells Can Enter a Quiescent G0 State, Although the Presence of G0 Tumor Cells Has Been Controversial

**Figure 3:**
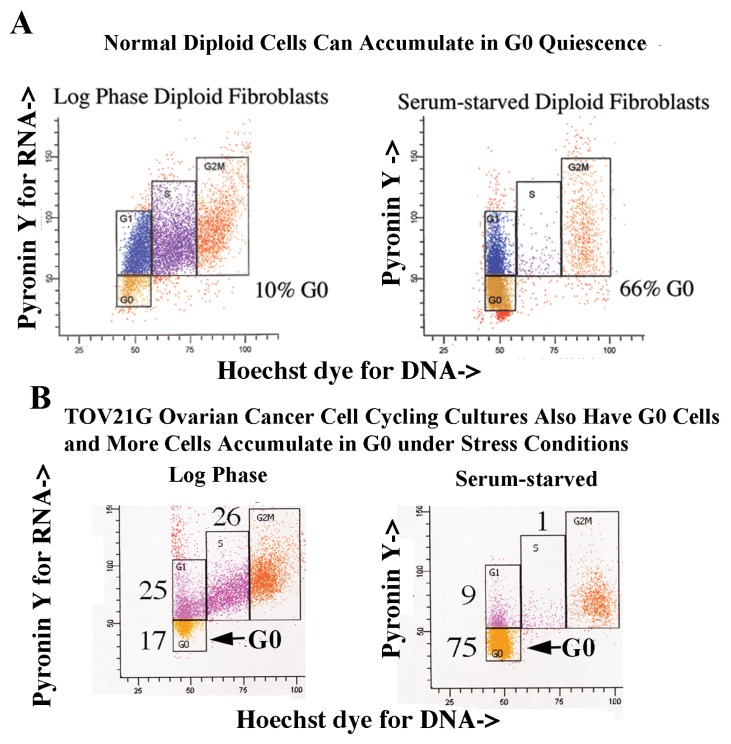
Normal diploid fibroblast cultures and TOV21G ovarian cancer cell cultures cycling in growth medium contain a small fraction of cells in G0 which increases when cells are grown in suboptimal, serum-limited growth conditions. (**A**, left) A log phase culture of the BJ strain of normal human diploid fibroblasts in growth medium was analyzed for cell cycle position by two parameter flow cytometry, first staining for DNA by Hoescht 33258, then staining for RNA using Pyronin Y. Boxes outline cells in G0, G1, S and G2 + M. G0 and G1 cells both have 2N DNA content so the G1 box is directly over the G0 box, with the G0 cells having less total RNA. (**A**, right) BJ normal human diploid fibroblasts were serum-starved for 72 h and then analyzed for cell cycle position by two parameter flow cytometry using the same gating and staining protocol as above. (**B**, left) TOV21G ovarian cancer culture cycling in growth medium has 17% G0 cells, while (**B**, right) cells serum-starved 4 days have 75% G0 cells.

Quiescent cells degrade their ribosomes allowing G0 cells to be identified by their lower RNA content than G1 cells, but 2N DNA content. Cellular DNA was stained with Hoechst 33258, Pyronin Y then added to bind to RNA, and both fluorochromes measured by two parameter flow cytometry [15]. The BJ strain of human normal diploid fibroblasts maintained in log phase growth had 10% of cells in G0 while serum-starvation placed the majority of cells (66%) in G0 (gold boxes in Figure 3A) [25]. TOV21G ovarian cancer cell cultures cycling in growth medium contain a small fraction of cells in G0, which increases when cells are serum-starved (Figure 3B).

**Figure 4:**
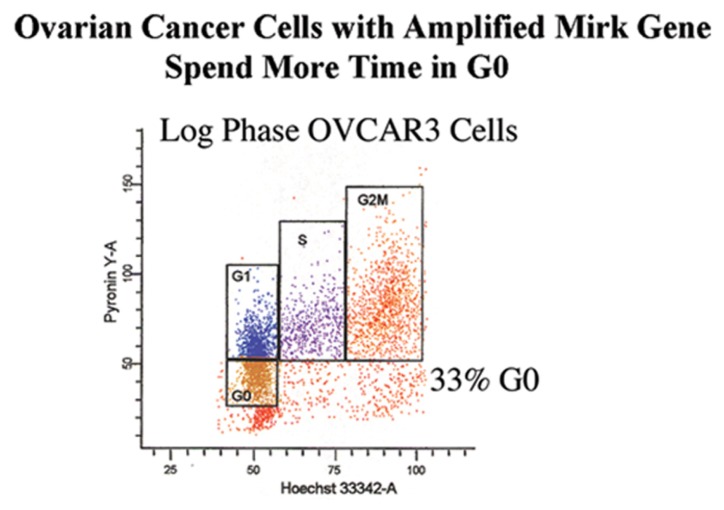
A log phase culture of OVCAR3 ovarian cancer cells was analyzed for cell cycle position by two parameter flow cytometry in identical fashion to the BJ fibroblasts in Figure 3, and showed a much higher fraction of G0 cells.

**Figure 5:**
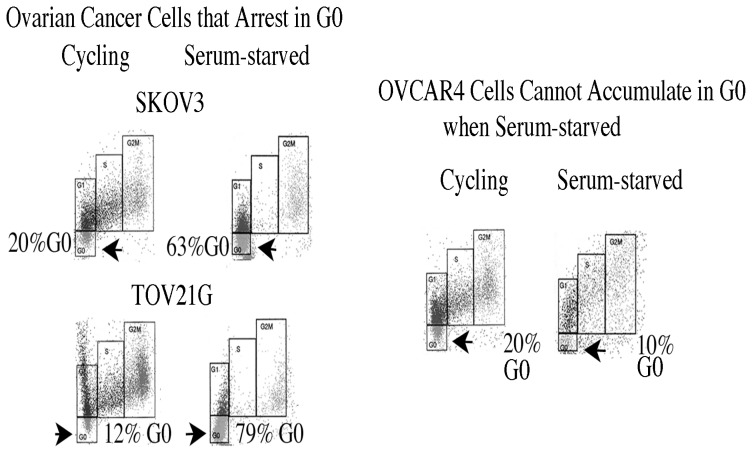
Ovarian cancer cell cultures contain a fraction in G0, which increased in some lines grown in suboptimal conditions. SKOV3, TOV21G, and OVCAR4 ovarian cancer cells were plated, and allowed to enter cycle by culture in growth medium (DMEM + 7% FBS) for 24 h, then switched to serum-free medium for 3 days before analysis for cell cycle position by two parameter flow cytometry. G0 cells are indicated by arrows.

Five ovarian cancer cell lines were assayed during log phase growth to determine whether any cells cycled into a G0 state even when the majority of cells were proliferating, using the same procedure for staining DNA and RNA used for normal diploid fibroblasts. Surprisingly, OVCAR3 (Figure 4), SKOV3, TOV21G, OVCAR4 (Figure 5) and OV90 ovarian cancer cell cultures each contained a fraction of cells in G0 [15]. The fraction of G0 cells averaged 17% ± 3% (SD) for SKOV3 cultures, 17% ± 4% for TOV21G cultures, 38% ± 2% for OVCAR3 cultures and 20% ± 2% for OVCAR4 and OV90 cultures. Reflecting their lower fraction of G0 cells, SKOV3 and TOV21G cultures grew over twice as fast as OVCAR3 cultures. Thus the entry of cells into G0 lowers the fraction of cycling cells within the culture, increasing the average doubling time.

### 2.8. G0 Is not a Permanent State

Quiescent G0 SKOV3 cells eventually re-entered the cell cycle when cultured in the presence of fresh nutrients showing that G0 arrest is transient. After switch to growth medium containing the mitotic inhibitor nocodazole, about 90% of the cells had exited the quiescent G0 state, traversed G1 and S and had been arrested in G2 + M by nodocazole, without the appearance of a sub-G1 peak of apoptotic cells [15].

### 2.9. G0 Is Induced by Various Suboptimal Culture Conditions, and not All Ovarian Cancers Can Enter a Reversible Quiescent State

TOV21G, SKOV3, OVCAR4, and OV90 cells were cultured for 3 days either in serum-free DMEM or maintained as log phase cultures in DMEM plus 7% FBS. Culture under serum-free conditions increased the fraction of G0 cells an average of 3.2 (± 0.2) fold for SKOV3 cultures and 6.5 (± 0.4) fold for TOV21G cultures, but did not increase the fraction of G0 cells in OVCAR4 or OV90 cultures (Figure 5, G0 cells indicated by arrows) [15].

### 2.10. Why didn’t OVCAR4 (Figure 5) or OV90 Cells Arrest Reversibly in G0, and did this Lack of Arrest Affect Their Viability?

The protein level of p130/Rb2 is elevated in quiescent cells where p130/Rb2 functions, but is lower in proliferating cells due to turnover [26], so an increase in p130/Rb2 levels should occur to facilitate the entry of ovarian cancer cells into G0. A time-course analysis showed that when TOV21G and SKOV3 cells were cultured in serum-free medium for 3 days and cells entered G0, the protein level of p130/Rb2 increased several-fold, while neither increase in p130/Rb2 nor accumulation in G0 occurred in OVCAR4 cells [15].

Immunohistochemical analysis by others revealed loss or decrease in expression of p130/Rb2 in about 40% of 45 resected human ovarian cancers assayed [27], so the OVCAR4 and OV90 lines may reflect this large subgroup of ovarian cancers with low p130/Rb2 expression.

### 2.11. Accumulation in G0 by Mirk Action Allows Ovarian Cancer Cells to Survive Suboptimal Culture Conditions

**Figure 6:**
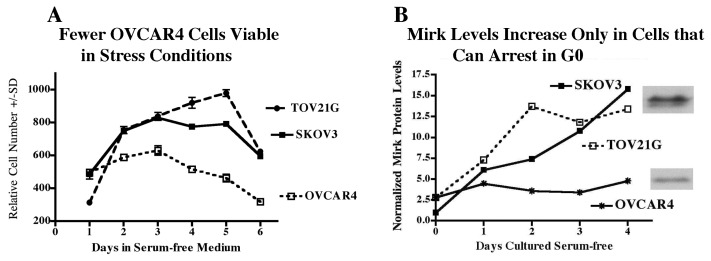
Cultures of OVCAR4 cells, which cannot accumulate in G0 under serum-free culture conditions, undergo more apoptosis and more cell death than cells that can arrest in G0, and do not accumulate Mirk protein. SKOV3, OVCAR4 and TOV21G cells were plated, and allowed to enter cycle by culture in growth medium for 24 h, then switched to serum-free medium. (**A**) Relative cell number was measured by the MTT assay, mean ± SD shown, *n* = 2. (**B**) Mirk protein levels normalized to actin.

SKOV3, TOV21G and OVCAR4 cells were cultured in serum-free conditions for up to 6 days (Figure 6). Measurement of cell viability by dye exclusion showed that about 60% of the OVCAR4 cells were nonviable and unable to exclude dye after 4 to 6 days of serum-free culture, compared with about 30% of SKOV3 and TOV21G cells [15]. These nonviable OVCAR4 cells then underwent apoptosis, reducing total cell numbers (Figure 6A). Loss of OVCAR4 cells in serum-free media was seen in multiple experiments and confirmed by showing that twice as many dead OVCAR4 cells as SKOV3 or TOV21G cells were detected by trypan blue exclusion (Figure 3c in [15]), and that OVCAR4 cultures contained far more cleaved PARP and cleaved caspase 3, markers of apoptosis (Figure 3b in [15]). Mirk protein levels increased 4 to 7 fold in SKOV3 and TOV21G cells, but not in OVCAR4 cells, cultured under these suboptimal growth conditions (Figure 6B), (Figure 2d in [15]). In time-course studies Mirk levels increased within 24 h of the switch to serum-free culture and then continued to increase. Thus an increase in level of Mirk protein was found only when ovarian cancer cells accumulated in G0 [15]. The rise in Mirk protein levels in these serum-free culture conditions only in TOV21G and SKOV3 cultures, but not in OVCAR4 cultures, again imply a role for Mirk in tumor cell survival.

Other suboptimal culture conditions, high cell density or low glucose DMEM, also led TOV21G and SKOV3 cells to accumulate in G0 and increased Mirk protein levels. Thus ovarian cancer cells that could enter a reversible quiescent arrest in G0 were more protected from suboptimal growth conditions than tumor cells that lacked this capacity. *In vivo*, such quiescent cells could re-enter the cell cycle under favorable clues from the microenvironment.

### 2.12. Ovarian Cancer Cells, not Normal Diploid Cells, Are Damaged by Mirk/dyrk1B Kinase Inhibition

Depletion of Mirk/dyrk1B led to increased cyclin D levels, an elevated reactive oxygen species content and loss of viability in cancer cells [2,8,19,28]. However, many normal cells *in vivo* are quiescent, and therefore, targeting a kinase found in quiescent cells might be problematic. To determine whether Mirk kinase inhibition might differentially target cancer cells, we first measured Mirk kinase activity by immunoprecipitating Mirk and then performing an *in vitro* kinase assay on the Mirk immunoprecipitates [28].

### 2.13. Mirk Kinase Activity Is Higher in Cancer Cells than Normal Cells

Mirk kinase activity was found to be at least 2-fold to 17-fold higher in ovarian cancer cells than in normal diploid cells, either normal ovarian epithelial cells in primary culture, or normal diploid fibroblast strains [28].

### 2.14. Effect of Mirk Kinase Inhibitors on CDK4/cyclin D Complexes, as a Measure of the Ability of Mirk Kinase Inhibition to Push Cells into Cycle from Quiescence

Pharmacological inhibition of Mirk/dyrk1B kinase increased cyclin D levels both in quiescent normal diploid cells and in quiescent CDKN2A-negative ovarian cancer cells, but led to more active CDK4/cyclin D complexes in quiescent ovarian cancer cells, because of the lack of CDK inhibitors [28]. Thus Mirk kinase inhibition should allow ovarian cancer cells with low expression of CDK inhibitors or overexpression of G1 cyclins to enter S phase prematurely.

**Figure 7:**
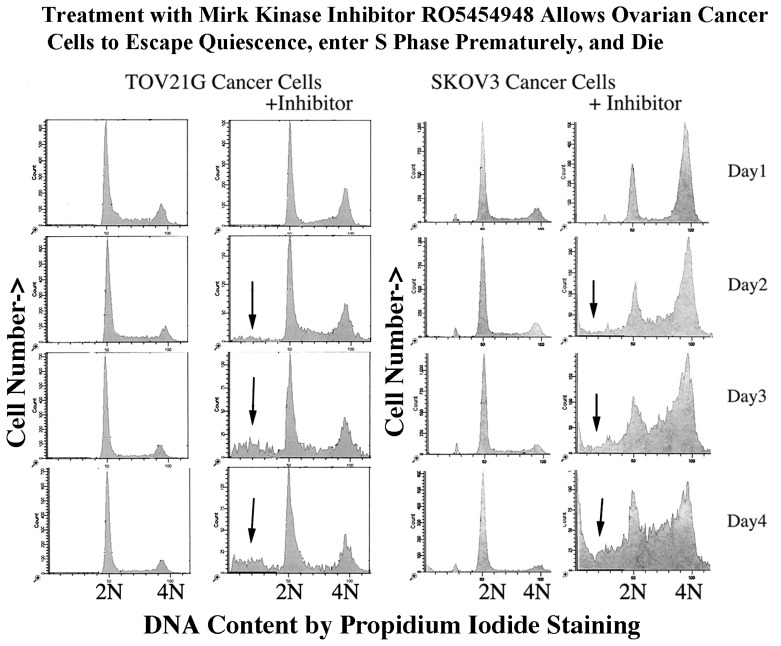
Mirk inhibitor RO5454948 induces cell cycle progression and sub-G0/G1cells in ovarian cancer cell cultures. Log phase TOV21G and SKOV3 ovarian cancer cells, both CDKN2A-negative, were plated at 5 × 10^5^ in 60 mm culture dishes and allowed to attach overnight. Media was changed to serum-free DMEM to induce quiescence, with TOV21G and SKOV3 cells treated with 0.5 μM and 0.75 μM, respectively, Mirk kinase inhibitor. DNA distributions were assayed by one parameter flow cytometry following staining with propidium iodide. Arrows indicate sub-G0/G1 apoptotic cells. One of duplicate experiments shown [28].

**Figure 8:**
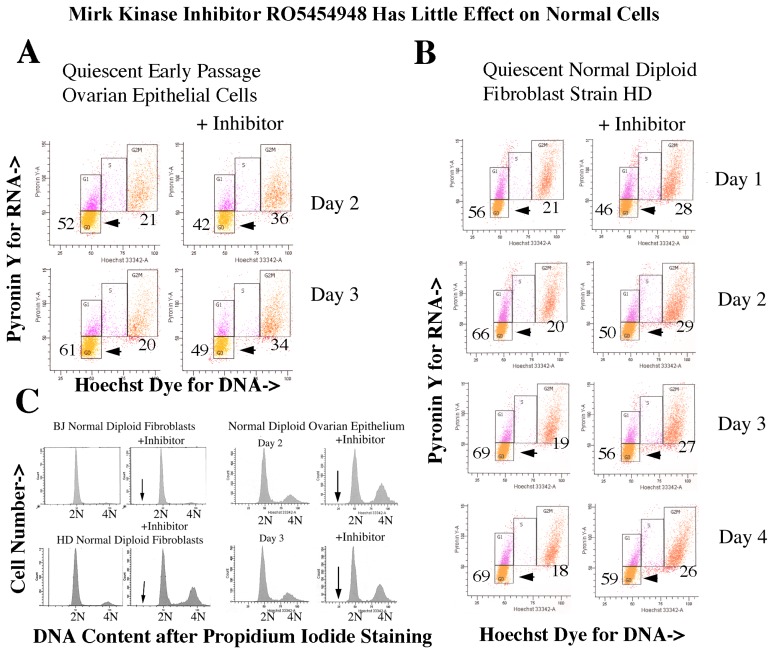
The Mirk kinase inhibitor RO5454948 has little effect cell cycling and viability of normal diploid cells. (**A**) Early passage normal human diploid ovarian epithelial cells were made quiescent by culture in serum-free ovarian epithelial cell medium (ScienCell), with one set treated with 0.5 μM of Mirk kinase inhibitor. G0, arrows. %G0 and %G2 + M cells are given after two-parameter flow cytometry. Similar data seen after 1 day treatment. (**B**) HD normal human diploid fibroblasts were made quiescent by culture in serum-free DMEM for 1 to 4 days, with one set treated with 0.5 μM of Mirk kinase inhibitor, and %G0 and %G2 + M cells given after two parameter flow cytometry. (**C**) HD and BJ strains of normal diploid fibroblasts and early passage normal human diploid ovarian epithelial cells were made quiescent by serum-starvation and treated with 0.5 μM of Mirk kinase inhibitor for 2 or 3 days, as noted. Cell cycle analysis was after propidium iodide staining [28]. Upright arrows indicate position of sub-G0/G1 apoptotic cells, but not present.

To test this hypothesis, the effect of Mirk kinase inhibitors on the cell cycle progression and induction of sub-G0/G1 apoptotic cells was analyzed by flow cytometry, using the more quantitative one-parameter measurements of DNA content, as well as the two parameter flow cytometry to detect G0 cells shown in Figures 3–5. Normal diploid cells and ovarian cancer cells were compared in this analysis (Figures 7 and 8).

Both TOV21G and SKOV3 cells were observed to slip out of quiescence into cycle when treated with the Mirk inhibitor at about their EC_50_ levels, as shown by an increased fraction of S and G2 + M cells (Figure 7). This was more pronounced in the p53null SKOV3 cells compared with the p53 wild-type TOV21G cells. However, sub-G0/G1 apoptotic cells were observed after 2–4 days of treatment of both CDKN2A-negative lines with the Mirk kinase inhibitor (arrows). By 4 days apoptotic cells comprised 15%–25% of the either tumor cell population. Thus CDKN2A-negative ovarian cancer cells treated with a Mirk kinase inhibitor escaped G0/G1 quiescence, entered cycle with high ROS levels and underwent apoptosis [28].

### 2.15. Many Normal Cells in the Body Are Quiescent, so We Questioned Whether Targeting Mirk might Allow Them to Escape Quiescence

Normal diploid ovarian epithelial cell cultures were made quiescent by culture in serum-free medium and treated with the Mirk inhibitor for 2 and 3 days (Figure 8). The major effect of Mirk kinase inhibition was to enable about 10%–12% of cells to leave G0 and to increase the G2 + M cell fraction from about 20% to about 35% (Figure 8). Few cells were seen in S phase. About 70% of cells in the HD strain of normal diploid dermal fibroblast quiescent cultures accumulated in G0 after 4 days of serum starvation, with about 10% fewer G0 cells in Mirk kinase inhibitor-treated parallel cultures. Mirk kinase inhibition moved about 8% more cells into G2 + M. Thus the Mirk kinase inhibitor slightly reduced the fraction of G0 quiescent diploid epithelial cells and fibroblasts, while the majority of the cells pushed into cycle accumulated in G2 + M. Apoptotic sub-G0/G1 cells were not detected [28],(Fig. 8C).

**Figure 9:**
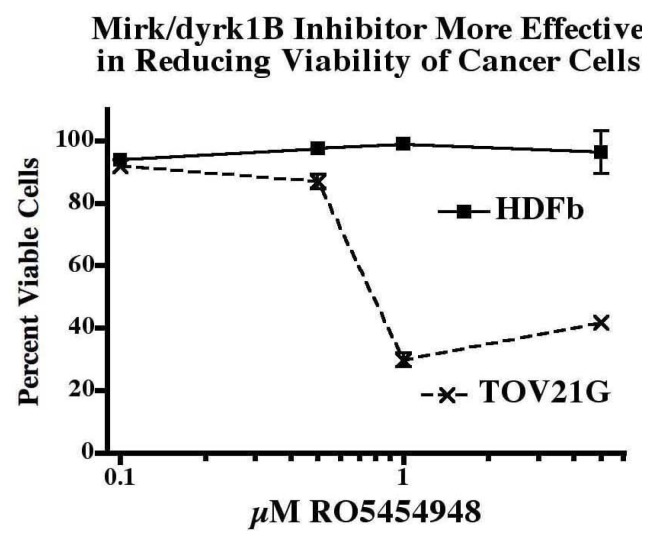
The relative decrease in viability induced by Mirk kinase inhibition in quiescent normal diploid cells and in cancer cells shown. The capacity of Mirk kinase inhibitor RO5454948 concentrations from 0.1 to 5 μM to kill HD normal human diploid fibroblasts (HDFb) and TOV21G cancer cells was compared by trypan blue exclusion. 5 × 10^4^ cells were plated per 6 well plate and maintained in DMEM + 0.2%FBS for 8 days. A mean of 315 cells was assayed for each data point (*n* = 2, ±SD if >5%). The control values were set to 100% [28].

As a result, Mirk kinase inhibited normal diploid cells were about 94% as viable as untreated controls, while Mirk kinase inhibition in quiescent ovarian cancer cells led a loss of about 70% of viable cells. This difference is quantitated in Figure 9 (below).

The ROS scavenger N-acetyl cysteine reduced both the amount of cleaved poly(ADP-ribose) polymerase (PARP) and the extent of cancer cell loss [28]. In contrast, normal cells were spared because of their expression of cyclin directed kinase inhibitors that blocked unregulated cycling. Quiescent early passage normal ovarian epithelial cells and two strains of quiescent normal diploid fibroblasts remained viable after the inhibition of Mirk/dyrk1B kinase, and the few cells that left G0/G1 quiescence were accumulated in G2+M, as seen in Figure 8[28]. Thus, inhibition of Mirk kinase targeted quiescent ovarian cancer cells.

### 2.16. Test of Mirk Kinase Inhibitor on Patient Ascites

Other investigators have shown that 5 of 5 ovarian cancer ascites samples from patients were quiescent, as judged by their about 85% G0/G1 content by flow cytometry, and low incorporation of BUdR [29]. These G0 cells regained the capacity to grow when cultured on adherent tissue culture plates, so their quiescence was reversible. Also, recent genomic analyses have identified p53 mutations in 96% of a survey of 489 high-grade serous ovarian adenocarcinomas [12]. Thus, ovarian cancer ascites might be the ideal tumor type to test the effect of Mirk kinase inhibition. In our own, ongoing studies with ovarian cancer ascites from patients, all have responded to either a Mirk kinase inhibitor alone, or in combination with another drug which increased Mirk expression levels.

## 3. Conclusions

Mirk is not an essential gene as embryonic knockout of Mirk/dyrk1B caused no evident phenotype in mice [30]. Thus pharmacological inhibition of Mirk kinase might have no deleterious effects on normal tissue. In fact our ongoing studies using experimental murine systems have shown that concentrations of a Mirk kinase inhibitor that induced apoptosis in ovarian cancer cells in culture and decreased the size of pancreatic cancer xenografts *in vivo*, did not inhibit mouse growth and caused no obvious decrease in mouse liveliness. What cancers might be selected for Mirk kinase inhibition? Candidates would be ovarian cancer cells that can achieve a reversible growth arrest in a quiescent state, perhaps the 60% with normal p130/Rb2 expression, unlike OVCAR4 cells (Figures 5 and 6). These candidate tumors would have low expression of CDK inhibitors (like SKOV3 and TOV21G cells in Figure 7) or elevated levels of G1 cyclins such as the 20% of high-grade serous adenocarcinomas with cyclin E amplification [12]. Mutation in p53, found in almost all of these tumors [12] so allowing limited stress induction of the p21 CDK inhibitor, could also potentiate response, similar to the greater response of SKOV3 cells than TOV21G cells with wild-type p53 (Figure 7).

## Figures and Tables

**Figure 10:**
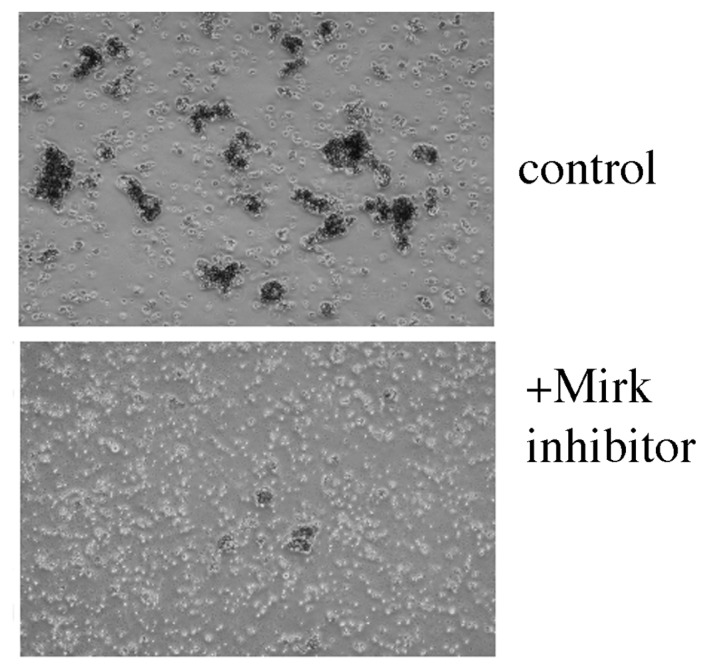
Mirk kinase inhibitor EHT5372 kills ovarian cancer ascites. 1 liter of ascites was centrifuged in conical tubes to pellet the malignant cells. The red blood cell layer was aspirated and the remaining tumor cells were resuspended in fresh medium, and aliquoted into multiple low attachment tissue culture dishes. The malignant cells formed into loose multicellular “spheroids”. After 24 h, the ascites were treated with Mirk/dyrk1B kinase inhibitor, and after 2 additional days the Mirk kinase inhibitor caused a 5-fold increase in the apoptosis marker cleaved PARP (not shown). Ovarian ascites are largely in a quiescent G0/G1 state characterized by high levels of the CDK inhibitor p27. The Mirk/dyrk1B kinase inhibitor reduced p27 levels, suggesting that cells escaped quiescence, as ovarian cancer cell lines do in culture (Figure 7). After 8 days in culture, the larger ascites spheroids were mostly broken down to single cells.
